# cl-dash: rapid configuration and deployment of Hadoop clusters for bioinformatics research in the cloud

**DOI:** 10.1093/bioinformatics/btv553

**Published:** 2015-10-01

**Authors:** Paul Hodor, Amandeep Chawla, Andrew Clark, Lauren Neal

**Affiliations:** Booz Allen Hamilton, Rockville, MD 20852, USA

## Abstract

**Summary**: One of the solutions proposed for addressing the challenge of the overwhelming abundance of genomic sequence and other biological data is the use of the Hadoop computing framework. Appropriate tools are needed to set up computational environments that facilitate research of novel bioinformatics methodology using Hadoop. Here, we present cl-dash, a complete starter kit for setting up such an environment. Configuring and deploying new Hadoop clusters can be done in minutes. Use of Amazon Web Services ensures no initial investment and minimal operation costs. Two sample bioinformatics applications help the researcher understand and learn the principles of implementing an algorithm using the MapReduce programming pattern.

**Availability and implementation**: Source code is available at https://bitbucket.org/booz-allen-sci-comp-team/cl-dash.git.

**Contact:** hodor_paul@bah.com

## 1 Introduction

The unprecedented abundance of sequence and other biological data generated in recent years has been straining traditional analysis approaches. A promising solution to the challenges of biological ‘big data’ is the Hadoop ecosystem of distributed computing tools combined with a cloud service. A Hadoop system runs on a cluster of arbitrary size consisting of commodity computers. It is based on a distributed file system and software implementing the MapReduce programming pattern. A growing number of applications of Hadoop/MapReduce in bioinformatics have been recently reported (reviewed by O’Driscoll *et al.*, 2013; Zhou *et al.*, 2013; Zou *et al.*, 2013).

Setting up a working Hadoop cluster remains a complex and time-consuming process. It can pose a considerable barrier to a bioinformatics researcher, who is interested to learn about this new technology and test it on their favorite problem. Some excellent deployment and cluster management tools already exist, but they fall short when considering the specific needs of a researcher primarily interested in a quick and easy setup for experimentation and benchmarking. For example, tools targeting enterprise-level clusters sacrifice simplicity for richness of features (e.g. Cloudera Manager, http://www.cloudera.com/content/cloudera/en/products-and-services/cloudera-enterprise/cloudera-manager.html and Apache AMBARI, http://ambari.apache.org/). Systems such as Cloudgene (Schönherr *et al.*, 2012) and CloudDOE (Chung *et al.*, 2014) have been specifically developed for bioinformatics applications. However, they focus on graphical user interfaces and ease of use for end users, and do not work well for benchmarking in cases where multiple clusters of different sizes and settings need to be quickly deployed and redeployed. Amazon Web Services (AWS) (http://aws.amazon.com/) offers the Elastic MapReduce service, which is easy to use, but has no built-in data persistence, and software customization is complex.

We set out to develop a tool for Hadoop cluster deployment and management, which specifically addresses the needs of bioinformaticians exploring the applicability of this technology to their research. Features of the tool that we considered critical included rapid and simple creation of new clusters of different sizes, flexible configuration and custom software installation, data persistence during intermittent usage and low cost of initial investment and during operation. Our implementation, called cl-dash, is a complete starter kit that allows quick setup of a Hadoop computing environment in a cloud environment. It is an ideal entry point for learning, prototyping and benchmarking of new algorithms. Two included sample applications help the user understand the principles of MapReduce programming as applied to bioinformatics.

## 2 Design and implementation

The core of cl-dash is a set of command-line tools that deploy and manage Hadoop clusters in the cloud environment provided by AWS. This commercial service has become popular for biomedical research (Fusaro *et al.*, 2011) due to its flexibility in implementing customized projects, ease of transition from small prototype to large production system, robust security and cost-effective operation.

**Fig. 1.**
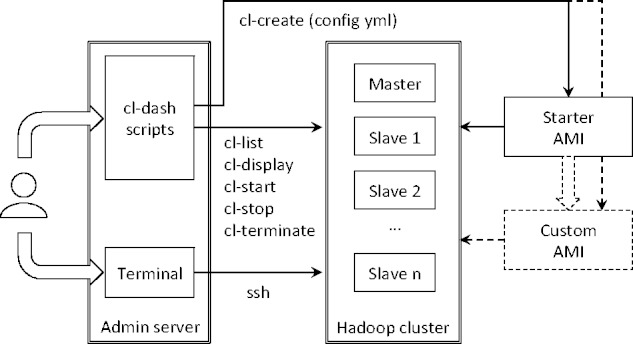
Architecture of a Hadoop cluster managed by cl-dash

Components of the cl-dash system, and how they interact with one another, are shown in Figure 1. A user creates and manages clusters from a generic ‘admin’ server, through a set of command-line tools, whose names begin with ‘cl-’ (hence the name of the system: ‘cl dash’). A new cluster can be created in minutes by providing a simple YAML configuration file (config.yml) and specifying an existing Amazon Machine Image (AMI). Once the Hadoop cluster is running, the user can log in through ssh and begin using it, or execute other cl-dash cluster management commands.

The cl-dash tools are Python scripts that use the boto library (http://boto.readthedocs.org) to interface with AWS. They can be run from any system that supports Python, such as a personal computer or a small server on AWS set aside for this purpose.

Six commands perform common cluster management tasks:

**cl-create**: launches a new cluster, having the name of the configuration file as a required parameter. The configuration file is in YAML format and specifies several variables that describe characteristics of the cluster, such as the number of and the size of computing nodes, the file path to the security key needed to access the cluster, metadata tags to identify the cluster and the identifier of the AMI to be used as a template for the master and slaves instances. The starter AMI that is provided with cl-dash is a specially configured Ubuntu system that has Hadoop and other software preloaded. Customized AMIs can be easily derived from the starter, and may be desirable in scenarios such as installing additional software (e.g. the Spark engine), increasing or reconfiguring storage, or changing global cluster parameters.
**cl-list**: shows all existing clusters by name and indicates their status, whether they are running or stopped.
**cl-display**: provides more information for a particular cluster. This command displays settings initially specified in the YAML configuration file, as well as public DNS and IP addresses for each cluster node.
**cl-start** and **cl-stop**: are used to start and stop an existing cluster on demand. These commands ensure the orderly turning on and off of software services such that files stored in the distributed file system persist and become available when the cluster is restarted. This mechanism is useful for conserving resources under the AWS pay-as-you-go pricing system.
**cl-terminate**: destroys a cluster that is no longer needed. All existing data are deleted and allocated resources are freed.

Creation of a new cluster involves the execution of a series of automated steps, which include launching and configuration of the compute nodes and starting up a series of services. Each step includes error checking, which facilitates troubleshooting in case of failures. The process of starting up an existing cluster is similar to new cluster creation, with the exception of instance provisioning and file system formatting, which only need to be executed once during cluster creation.

Users interact with a running cluster by logging in through ssh. Although it is possible to log into any node, typically one would directly interact only with the master node. User code, input and output data can be transferred through scp to and from the local file system of the master node.

## 3 Sample applications

The starter AMI provided with cl-dash includes two sample MapReduce applications. They are intended for bioinformaticians new to the Hadoop ecosystem who are interested in understanding how they could apply this technology in their own research. Both applications are ready to run, both in a traditional, sequential version, and in a MapReduce version.

The first application determines amino acid frequencies in the human proteome. It is a demonstration of how a simple problem can be framed in MapReduce terms. The task is subdivided into two short Perl scripts, a mapper and a reducer. The mapper splits the input sequences into individual amino acids, and the reducer counts the number of amino acids of the same type.

The second application performs a genome-wide association study (GWAS) analysis. It shows how an existing, single-threaded software can be wrapped into MapReduce to increase performance. An unmodified version of the PLINK executable is used for GWAS. A pair of Perl scripts constitute the wrapper and ensure parallelized execution of PLINK and conflation of output.

## 4 Discussion

We have developed cl-dash as an easy-to-use Hadoop deployment method to facilitate development and benchmarking of bioinformatics applications that use Hadoop technology. As a learning system, it offers a straightforward introduction to the student. With minimal investment of time and money, a working cluster can be set up and sample applications can be run and analyzed. Used as a research system, it allows prototyping on a small cluster, followed by benchmarking on multiple clusters of widely varying sizes. The scaling up and down of computing capacity is a built-in feature of the system and is available with no up-front costs.

One apparent limitation of the system is its dependence on the commercial AWS service. However, the benefits of low cost, shallow learning curve and flexibility make AWS a prime choice for the common use cases described above.

We have routinely used cl-dash in our own research into developing novel big data tools for bioinformatics. For example, we have explored the feasibility of the Hadoop framework to store, manage and analyze large-scale human genomic variation data. We have found the system to be ideally suited for a highly dynamic work environment focused on experimentation.

*Conflict of Interest*: none declared.
